# Examining Personal and Media Factors Associated with Attitude towards Genetically Modified Foods among University Students in Kunming, China

**DOI:** 10.3390/ijerph16234613

**Published:** 2019-11-20

**Authors:** Li Li, John Robert Bautista

**Affiliations:** 1 School of Journalism, Yunnan University, Kunming 650000, China; 2 School of Information, The University of Texas at Austin, Austin, TX 78701, USA; jrbautista@utexas.edu; 3 Center for Health Communication, Moody College of Communication, The University of Texas at Austin, Austin, TX 78712, USA

**Keywords:** attitude, food neophobia, genetically modified foods, media attention, media system dependency theory

## Abstract

Guided by the theory of reasoned action and media system dependency theory, this study examined attitude towards genetically modified foods (GMF) among university students in Kunming, China, as well as personal and media factors related to such attitude. Data were collected from an online survey with 467 university students across eight universities in Kunming. Results showed that attitude towards GMF were more negative than positive. Moreover, food neophobia and media attention were negatively associated with attitude towards GMF. In contrast, perceived benefit was positively associated with attitude towards GMF. Although the interaction between media dependency and media attention was significant, simple slope analysis confirmed that the interaction slopes were nonsignificant, suggesting that media attention did not moderate the association between media dependency and attitude towards GMF in this study. Theoretical and practical implications are discussed.

## 1. Introduction

Genetically modified foods (GMF) refer to foods that are derived from organisms (i.e., plants or animals) whose genetic materials have been modified in ways that do not occur naturally [1]. Some of the reasons for altering the genetic makeup of such organisms are to reduce crop damage (e.g., damage caused by viruses, bacteria, insects, and pesticides), increase the resulting food’s nutritional value, and increase food production [2]. Aside from improving yield and nutritional value, such modification is also a means to reduce food prices and combat malnutrition, especially in developing countries, where the population outweighs local food production [3,4].

Although GMF is a viable solution to meet the required food supply for a continuously growing global population, it faces several issues considering that it is a perceived threat to biodiversity, environment, and human health [3]. This makes GMF a prime source of heated public debates that have implications on consumers’ attitude towards GMF. Scholars have noted that such debates have placed GMF on negative media limelight, and this contributes to the public’s negative attitude towards GMF [5,6]. Thus, improving consumers’ attitude towards GMF and encouraging GMF purchase and consumption decisions in the long term have become major challenges for the authorities [7]. For this reason, the role of media in the formation of attitude towards GMF must be investigated. 

The present study examined personal and media factors associated with attitude towards GMF in China. Guided by the theory of reasoned action (TRA) [8], this study incorporated personal factors, such as food neophobia, factual knowledge, perceived familiarity, and perceived benefits, to predict attitude towards GMF. More importantly, this study took into account the role of media in GMF attitude formation by incorporating media factors, such as media attention and media dependency, based on the media system dependency (MSD) theory [9]. China serves as an ideal context for this study because, for the past two decades, its government has invested heavily in GMF research and development to improve its food security [5]. Moreover, China has the highest population in the world, and ensuring its food supply through GMF is a matter of national interest despite the issues associated with it [10]. 

Currently, several research gaps must be addressed regarding the role of media in the formation of attitude towards GMF. First, most published studies are from Western countries (e.g., [7,11,12]). Second, few studies in China have used theories to examine factors associated with public perceptions of GMF (e.g., [13,14,15]). Finally, within these theory-based studies, none has incorporated media factors even though genetically modified organisms and food have been a widely covered news topic globally [4,16,17] and in China [15,18,19]. 

To overcome these research gaps, the present study provides theoretical insights on attitude towards GMF in China, including personal and media factors associated with such attitude. While the study contributes to theory, it also provides practical insights that stakeholders (e.g., GMF food producers and distributors) and news organizations can use to better communicate GMF-related information to the public.

## 2. Literature Review

### 2.1. Attitude towards GMF

The controversial nature of GMF makes it a polarizing issue among consumers. As Zhang and Liu [20] noted, consumer attitude towards GMF may range from positive (acceptance and optimism) to negative (rejection and pessimism). Such polarization has been documented by research works published in the past 20 years, with people from Australia [21], Europe [22,23,24,25,26], New Zealand [27], and Singapore [28] found to be less supportive of GMF, while those in the US [24], Japan [29], and South Africa [30] are supportive of it. 

In China, Cui and Shoemaker [5] summarized studies from 2002 to 2016 and found that attitude towards GMF there had shifted through the years. Specifically, studies from 2002 to 2011 indicated general support for GMF, whereas studies from 2012 to 2016 indicated general opposition for GMF. This trend seems to be in contrast with another study in China that conducted surveys in 2007, 2008, and 2010, wherein most of the respondents supported GMF development and large-scale cultivation of genetically modified crops [31]. Based on the abovementioned studies, it was interesting for us to determine whether the prevailing attitude towards GMF is positive or negative. Therefore, this study asked the following research question: 

RQ1: What is the respondents’ attitude towards GMF?

### 2.2. Personal Factors

The TRA [8] asserts that individuals form beliefs about an object, and this can automatically and simultaneously result in a formed attitude towards that object. Accordingly, attitude towards GMF can be predicted based on personal beliefs that consumers hold about GMF [20,32]. Furthermore, TRA is applicable in this study as it has been used as a framework to link personal factors with attitude towards GMF (e.g., [27,33]. In this study, some of the key personal factors that might be associated with attitude towards GMF were considered, including food neophobia, perceived benefit, factual knowledge, and perceived familiarity. 

#### 2.2.1. Food Neophobia

Food neophobia is an inherent adaptive personality trait, which refers to the rejection of novel or unknown food [34]. As GMF is based on genetic engineering techniques that may be difficult for most consumers to understand, there is a natural tendency for consumers to fear GMF consumption [32]. Aside from the fear of the unknown due to lack of information, consumers may also fear GMF due to several concerns, such as food irradiation, food hazards, and unnaturalness [35]. Conceptually, it can be inferred that food neophobia might result in a negative attitude towards GMF.

Some studies provide clues that link food neophobia with attitude towards GMF and other related factors. For instance, Bäckström et al. [36] found that food neophobia was negatively associated with willingness to consume GMF, such as snails and passion fruit. Moreover, Bredahl’s [37] qualitative work involving participants from the UK, Denmark, Germany, and Italy showed that participants who expressed food neophobia tended to have a negative attitude towards GMF. Similarly, a follow-up study by Bredahl [38] in the same European countries showed that food neophobia negatively influenced attitude towards genetic modification in food production through elevated perceptions of risks. Based on conceptual and empirical considerations, this study hypothesized the following:

**Hypothesis** **1** **(H1).** *Food neophobia is negatively associated with attitude towards GMF.*

#### 2.2.2. Perceived Benefits

New food technologies come with both benefits and concerns, and their commercial success occurs when the benefits outweigh the concerns [39,40]. As a by-product of genetic engineering (a new food technology), there will always be benefits and concerns with the production and consumption of GMF [35,39]. Nonetheless, global agencies, such as the World Health Organization (WHO) [2], take measures to ensure that internationally marketed GMF adhere to standards and are safe for consumption. This is based on the premise that GMF offer greater benefits than traditional food sources, such as additional nutrients, reduced allergens, and efficient food production [2]. Thus, it can be implied that greater perceptions of GMF benefit can improve consumer attitude towards GMF.

Previous works have indicated an association between perceived benefits and attitude towards GMF. Early research in Switzerland, for example, indicated that perceived benefit was positively associated with acceptance of gene technology for food production [41]. Studies in China [15] and Taiwan [32] suggested that perceived benefit had a positive association with attitude towards GMF. Moreover, a study in the US found that perceived benefit was positively associated with attitude towards GMF among both millennial and nonmillennial consumers, although the association was higher in the latter [11]. Similarly, a survey in Italy showed that perceived family and health benefits had a positive association with attitude towards genetic modification for food production [10]. Based on conceptual and empirical considerations, this study hypothesized the following:

**Hypothesis** **2** **(H2).** *Perceived benefit is positively associated with attitude towards GMF.*

#### 2.2.3. Factual Knowledge

The TRA [8] argues that one of the components of attitude formation includes the amount of knowledge that a person has about a certain object. Although there are several ways of conceptualizing knowledge, science communication scholars have recently used factual knowledge when assessing the extent of knowledge possessed by an individual about a scientific topic (e.g., [42,43,44]). “Factual knowledge” refers to concrete information and facts stored in an individual’s schema [43]. Other studies often label factual knowledge as objective knowledge (e.g., [7,15]). Such knowledge is often assessed by asking respondents to answer a series of true or false questions and computing the number of correct answers [42,44]. In such a test, a higher score denotes greater factual knowledge and is a manifestation of an individual’s ability to recall and understand facts related to a topic of interest. To date, the level of factual knowledge that consumers have on GMF in China is unclear. However, scholars suggest that factual knowledge of biotechnology and GMF is quite low in the US [40] and Belgium [7]. 

On the one hand, some previous studies have suggested a link between factual knowledge and attitude towards GMF. For instance, a study in China found that objective knowledge (i.e., factual knowledge) regarding the benefits and risks of GMF was positively associated with attitude towards GMF [15]. Moreover, a study in Belgium showed that objective knowledge was positively related to willingness to eat genetically modified crops among secondary school students [7]. On the other hand, another research reported that objective knowledge was positively associated with attitude towards consumption of organic vegetables [45]. Based on conceptual and empirical considerations, this study hypothesized the following:

**Hypothesis** **3** **(H3).** *Factual knowledge is positively associated with attitude towards GMF.*

#### 2.2.4. Perceived Familiarity

Perceived familiarity refers to the subjective idea of how much individuals know about a certain object or event regardless of their actual knowledge of it [46,47]. In contrast to factual knowledge, perceived familiarity is the degree of one’s awareness of an issue. As such, it is also referred to in scholarly literature as “subjective knowledge” [7,45,48]. Aside from factual knowledge, perceived familiarity with GMF must also be studied because consumers are relatively aware of GMF but may not have high levels of objective knowledge about the technology behind it [7,40]. This is consistent with a recent study advocating for the differentiation between factual knowledge and perceived familiarity as sources of scientific knowledge, particularly on GMF [49]. Considering that perceived familiarity is a source of knowledge and scientific understanding [47,49], it can be inferred that this variable can influence consumer attitude towards GMF. 

Relevant research supports an association between perceived familiarity (or subjective knowledge) and attitude towards food products. For instance, a study in Belgium found that subjective knowledge was positively related to willingness to eat genetically modified crops among secondary school children [7]. Moreover, a study among US consumers showed that subjective knowledge of Korean food was positively associated with attitude towards Korean food [50]. Likewise, subjective knowledge was found to be positively associated with attitude towards consumption of organic vegetables in [45]. Based on conceptual and empirical considerations, this study hypothesized the following:

**Hypothesis** **4** **(H4).** *Perceived familiarity is positively associated with attitude towards GMF.*

### 2.3. Media Factors

The MSD theory posits that an individual’s dependence to attain an outcome relies on the resources gathered from the media [9]. While this theory focuses on the interactions of the media with the macro- (social systems), meso- (groups and organizations), and micro- (audiences) levels [51], the focus of the current study is on the influence of media at the micro-level. Following the premise wherein the media has micro level (i.e., audience level) influences, it can be inferred that certain outcomes, such as attitude towards an issue, are associated with certain media factors [51]. In the literature (e.g., [51,52,53]), media attention and media dependency are two media factors that are potentially associated with attitude towards GMF.

#### 2.3.1. Media Attention

Media attention is defined as the level of conscious attention that individuals dedicate to particular types of media messages [54]. In this study, media attention refers to respondents’ attention to GMF news received via various media platforms, such as television, newspaper, internet, and social media. Some models of information processing and persuasion, such as the elaboration likelihood model [55], propose that attention to message content is a necessary condition for persuasive effects. Previous studies have also shown that mass media can cultivate and shape the perceptions and beliefs of individuals who are immersed in the media system [56,57]. Through exposure to different framed contents in the media, people receive information about different aspects of social issues and, consequently, form a diverse attitude towards these [58,59]. For instance, Chen [60] found that attention to food scandal-related news delivered via traditional and new media negatively influenced individuals’ attitude towards consuming food with additives, which in turn further influenced their intention to avoid consuming foods with additives.

Recently, with the heated debates about GMF among Chinese politicians, activists, and consumers, news messages on various media platforms have tended to lean toward the negative side [5]. This may contribute to the public’s overall negative attitude towards GMF. As such, we expect that the more attention people pay to GMF on various media platforms, the more likely they will be to hold a negative attitude towards it. Based on conceptual and empirical considerations, this study hypothesized the following:

**Hypothesis** **5** **(H5).** *Media attention is negatively associated with attitude towards GMF.*

#### 2.3.2. Media Dependency

Media dependency, which can motivate individuals’ attitude change, is proposed to be another imperative media factor affecting people’s attitude towards GMF. “Media dependency” is defined as the extent to which people rely on media to achieve goals [9,61]. Based on the MSD theory, if an individual heavily relies on media to fulfill his/her needs, this means that the media gradually occupies a more important role and has a stronger impact on that person [62]. In other words, the greater the dependence on media, the greater its influence to change or modify a person’s attitude and behavior. Ho et al. [53] argued that media attention alone is insufficient to model the effects of media on people’s attitudes and behaviors. Some people may pay attention to the media but not feel that it can influence their attitude towards certain issues. Thus, we must consider people’s media dependency as a potential factor that can affect their attitude towards GMF.

Previous studies have shown a significant relationship between media dependency and attitude formation. For instance, a controlled field experiment conducted by Ball-Rokeach et al. [63] found that a higher degree of television dependency led to a greater selective exposure to specific dependency-relevant television content and, consequently, to more favorable postexposure responses in terms of values, attitudes, and beliefs concerning racism, sexism, and environmental pollution. As news messages related to GMF on Chinese media platforms tend to be negative due to heated discussions among several stakeholders [5], it can be inferred that individuals who are dependent on media to understand GMF are more likely to hold a negative attitude towards it. Based on conceptual and empirical considerations, this study hypothesized the following:

**Hypothesis** **6** **(H6).** *H6: Media dependency is negatively associated with attitude towards GMF.*

Moreover, the MSD theory suggests that when media dependency is intensified because of the increased attention during media exposure, the media effects on individuals will also be stronger [63]. A study supported this argument by reporting that the relationship between media dependency and green-buying intention were likely to increase for individuals who paid less attention to media than for those who paid more attention [53]. Based on this previous work, we proposed the following research question regarding the interaction of media attention and media dependency in predicting attitude towards GMF:

RQ2: Does media attention moderate the association between media dependency and attitude towards GMF?

## 3. Methods

### 3.1. Respondent Selection and Profiles

This study gathered survey data from university students in Kunming, a medium-sized city in Southwest China, in October 2017. As it is a preliminary study of public attitude towards GMF, we utilized purposive and snowball sampling techniques to recruit respondents. A URL link for the survey was sent to potential respondents via WeChat. Only university students were eligible to answer the survey. Potential respondents were also asked to invite their classmates and friends who were also university students in Kunming to take the survey. 

The study used the Declaration of Helsinki as a basis for conducting ethical research on human subjects. Accordingly, respondents provided consent before starting the survey, and no personally identifiable information was collected to ensure anonymity. The questionnaire used in the online survey was initially drafted in English and later translated into Mandarin. 

The data collection yielded 467 valid respondents with no missing data. The respondents came from eight universities in Kunming, China. Most were female (61%), and their average age was 20.76 years (standard deviation (SD) = 4.02). Moreover, a majority were undergraduate students (84.6%) enrolled in humanities (48.6%), followed by those in the science and engineering (28.5%) and social sciences (19.1%) programs.

### 3.2. Measures

Attitude towards GMF (mean (M) = 2.75, SD = 0.73, Spearman–Brown coefficient = 0.72). Attitude towards GMF was used as the dependent variable for this study and was measured using two items adapted from Maes et al. [7]: “Genetically modified food is unhealthy” (reverse coded) and “Applying gene technology in food production is dangerous” (reverse coded). Although these items were considered under perceived risk in Maes et al. [7], in the study’s context in China, such items reflect general attitudes that are prevalent (i.e., attitudes where GMF is considered unhealthy and dangerous) [5]. Responses were gathered using a 5-point Likert scale with scores that ranged from 1 (strongly disagree) to 5 (strongly agree). As the items were reverse coded, a higher score indicated a positive attitude towards GMF.

Food neophobia (M = 2.97, SD = 0.56, Cronbach’s α = 0.71). Food neophobia was measured using three items adapted from Maes et al. [7], namely, “I am afraid to eat things I have never had before” and “I do not trust new foods”. Responses were based on a 5-point Likert scale with scores that ranged from 1 (strongly disagree) to 5 (strongly agree). A higher score indicated high food neophobia.

Perceived benefit (M = 2.96, SD = 0.63, Cronbach’s α = 0.86). Perceived benefit was measured using eight items adapted from Zhu and Xie [15]. Sample items included the following: “Applying gene technology in food production will reduce the price of food products” and “Genetically modified food products are better quality foodstuffs than other food products”. Responses were based on a 5-point Likert scale with scores that ranged from 1 (strongly disagree) to 5 (strongly agree). A higher score indicated a high level of perceived benefit of GMF.

Factual knowledge (M = 5.66, SD = 1.16). Factual knowledge regarding GMF was measured using eight statements adapted from Ghasemi et al. [64]. Respondents were asked to answer each item as either true or false. The responses were coded as “1” for correct answers or “0” for incorrect answers. The questions were composed of a mix of easy and difficult statements. The knowledge statements, with the correct answers in brackets, were as follows: (i) “GM crops are the same as conventionally cross-bred crops” (false); (ii) “GM crops are the same as organic crops” (false); (iii) “GM crops are produced by taking genes from plant species and transferring them into plants” (true); (iv) “Non-GM foods do not contain genes, while GM foods do” (true); (v) “It is impossible to transfer animals’ genes into plants” (false); (vi) “Tomato modified with fish genes would taste fishy” (false); (vii) “GM foods can be distinguished from non-GM foods” (false); and (viii) “All processed foods are made using GM products” (false). A higher score indicated high factual knowledge about GMF.

Perceived familiarity (M = 4.40, SD = 0.87). Perceived familiarity was a single item question adapted from Yang et al. [44]: “On a ten-point scale with 1 being nothing at all and 10 being very much, please tell me which number between 1 and 10 best represents how much you have heard, read, or seen about GMF?” A higher score indicated high perceived familiarity with GMF.

Media attention (M = 2.68, SD = 0.64, Cronbach’s α = 0.83). Media attention was measured using five items adapted from Lin et al. [65]. The items asked respondents to indicate how much attention they gave to GMF messages delivered via newspaper, television, radio, internet, and social media (1 = no attention at all, 5 = very close attention). A higher score indicated high media attention to GMF.

Media dependency (M = 3.66, SD = 0.59, Cronbach’s α = 0.80). Media dependency was measured using four items adapted from Ho et al. [53]. Sample items included the following: “Getting information from the news media is helpful for me to find out about GMF” and “Getting information from the news media is helpful for me to figure out how to deal with GMF”. Respondents were asked to rate their agreement using a 5-point Likert scale (1 = strongly disagree, 5 = strongly agree). A higher score indicated high media dependency for GMF information.

Control variables. The effects of age (M = 20.69, SD = 4.02) and gender (female = 61%) were controlled in the analysis.

### 3.3. Data Analysis

IBM SPSS Statistics 21 (IBM, New York, NY, USA) was used to analyze the data. Aside from descriptive statistics (e.g., mean, standard deviation, percentage), hierarchical regression analysis was performed to examine the association between the study’s independent and dependent variables. Attitude towards GMF was entered as the dependent variable, and several independent variables were entered in blocks. Control variables (i.e., age and gender) were entered in the first block, followed by personal factors (i.e., food neophobia, perceived benefit, factual knowledge, and perceived familiarity) in the second block, media factors (i.e., media attention and media dependency) in the third block, and interaction variables (i.e., media attention × media dependency) in the final block. 

To examine whether media attention moderates the association between media dependency and attitude towards GMF, an interaction term was created by multiplying the mean-centered values of the respective main effect variables to reduce potential multicollinearity problems [66]. Zero-order bivariate correlation analysis was also conducted before running the regression analysis.

## 4. Results

### 4.1. Attitude towards GMF

RQ1 asked about the respondents’ attitude towards GMF. Results showed that the respondents had an overall negative attitude towards GMF based on the average of the two attitude items (M = 2.75, SD = 0.73). This was evidenced by a mean score of less than 3 (the items were reversed, so a score of less than 3 indicates a negative attitude). A close inspection of the mean scores of the items “Genetically modified food is unhealthy” (M = 2.71, SD = 0.81) and “Applying gene technology in food production is dangerous” (M = 2.79, SD = 0.85) indicated such negative attitudes. 

### 4.2. Factors Associated with Attitude towards GMF

Table 1 presents the results of the hierarchical regression analysis that show factors associated with attitude towards GMF. Overall, the independent variables were able to explain 33.4% of the attitude towards GMF’s variance.

After controlling for the effects of age (β = –0.08, *p* = 0.06) and gender (β = –0.05, *p* = 0.19), results showed that two of the four personal factors were associated with attitude towards GMF. Specifically, food neophobia (β = −0.09, *p* = 0.03) was negatively associated with attitude towards GMF, whereas perceived benefit (β = 0.54, *p* < 0.001) was positively associated with it. This supported H1 and H2. In contrast, H3 and H4 were rejected because factual knowledge (β = −0.03, *p* = 0.52) and perceived familiarity (β = 0.07, *p* = 0.08) were both not associated with attitude towards GMF. Finally, media attention (β = −0.09, *p* = 0.04) was negatively associated with attitude towards GMF, whereas media dependency was not associated with it (β = 0.02, *p* = 0.67). Therefore, these results supported H5 but not H6. 

### 4.3. Interaction of Media Attention and Media Dependency

RQ2 asked whether media attention moderates the association between media dependency and attitude towards GMF. Based on Table 1, media attention moderated the association between media dependency and attitude towards GMF (β = −0.09, *p* = 0.02). To further examine the interaction effect, we used Hayes’ PROCESS Macro version 3.4 (model 1) [67] to perform a simple slope analysis, which would allow us to determine the conditional effect of media attention between the association of media dependency and attitude towards GMF and whether such effects were statistically significant. While we found an interaction effect in Table 1, results shown in Table 2 suggest that such interactions were nonsignificant. Hence, media attention did not moderate the association between media dependency and attitude towards GMF.

## 5. Discussion

Based on a survey conducted among 467 university students in Kunming, China, this study sheds light on recent attitude towards GMF in the country as well as the personal and media factors that are associated with such attitude. The following paragraphs provide a discussion of the key findings of the study.

First, the study found that the respondents had an overall negative attitude towards GMF. Such a finding is consistent with that of Cui and Shoemaker [5] in China, where most respondents were found to have a more negative attitude towards GMF in recent years. A potential reason for such a negative attitude is that mass media in China frequently discusses GMF issues in a negative way [5]. On the other hand, the finding contradicts the study of Han et al. [31], which reported that more people supported GMF. Nonetheless, our results should be interpreted with caution since our sample was derived using nonprobability sampling that may not reflect the target population’s prevalent attitude towards GMF [68]. 

Second, although the results provide support to TRA by showing that beliefs can affect attitude towards an object, not all personal beliefs were found to be associated with attitude towards GMF in this study. Specifically, perceived benefit and food neophobia were associated with such attitude but factual knowledge and perceived familiarity were not. These findings are consistent with Rose et al.’s [49] recent study, where food consciousness—a construct similar to food neophobia—was shown to predict negative attitude towards GMF, while both types of knowledge did not. Such findings are also reminiscent of Hossain and Onyango’s study [69], where knowledge of science was reported not to predict acceptance of GMF. Although perceived benefit and food neophobia represent both ends of a spectrum, the former had a greater association with attitude towards GMF than the latter in this study. This is consistent with previous works [36,37,38], where the negative effect of food neophobia on attitude towards GMF was minimal compared to the positive effect of perceived benefit. The role of perceived benefit as a strong predictor among other predictors of attitude towards GMF is also supported by the study of Chen and Li [32]. Based on the results, we can argue that despite the presence of food neophobia and regardless of consumers’ prior knowledge (i.e., factual knowledge and perceived familiarity), communicating the benefits of GMF might be a strategic move in improving people’s attitude towards GMF. 

Third, this study found that media attention, but not media dependency, was negatively associated with attitude towards GMF. Moreover, we also did not find an interaction between these variables. Nonetheless, the results support the notion that the media can influence people’s attitude towards issues of public concern [58,59]. The more attention the respondents paid to GMF delivered through various media platforms, the more likely they were to hold a negative attitude towards it. This result is consistent with previous research (e.g., [57,60]) and models of information processing and persuasion, such as the elaboration likelihood model [55], which proposes that attention to message content is a necessary condition for persuasive effects. As mass media in China frequently discusses GMF issues negatively due to heated discussions among Chinese politicians, activists, and consumers [5], it is somewhat expected that attention to GMF in various media platforms negatively predicts people’s attitude towards GMF.

## 6. Implications

### 6.1. Theoretical Implications

The study has several theoretical implications. First, it adds to our theoretical understanding of the factors associated with the formation of attitude towards GMF in China. More importantly, it adds to the relatively few studies that have used theories to examine consumer perceptions of GMF in China (e.g., [13,14]). The study also highlights the usefulness of MSD theory when predicting attitude towards GMF. Although the MSD theory has been used to examine food issues (e.g., [33,70,71]), to the best of our knowledge, the current study is one of the first to have applied such theory to examine attitude towards GMF. While the results showed that media dependency was a nonsignificant predictor of attitude towards GMF in this study, it also showed that an association occurred when media dependency interacted with media attention. Although the simple slope analysis revealed that such interaction yielded statistically nonsignificant slopes, future studies should further explore the moderating effect of media attention on health and environmental issues in conjunction with individuals’ level of media dependency. 

### 6.2. Practical Implications

In terms of practical implications, the study suggests that stakeholders involved in the production and dissemination of GMF should be aware of consumers’ attitude towards GMF considering that scholars have established that such perceptions can affect their purchase and consumption behaviors related to GMF [7,14]. As the present study showed that the respondents had an overall negative attitude towards GMF, stakeholders should continuously exert efforts to better communicate the benefits and safety of GMF consumption. Moreover, the study also provides insights to news organizations regarding the influence of the media on public attitude towards GMF. Although news organizations should deliver balanced coverage of positive and negative news about GMF, they should also proactively incorporate scientific facts in their reports and weed out exaggerated and fake news that can undermine the formation of an overall positive public attitude towards GMF [5].

## 7. Limitations and Future Research Directions

Although this study contributes knowledge on factors associated with attitude towards GMF in China, some limitations should be acknowledged. First, the sample only included university students from one city in China. As argued by Honkanen and Verplanken [72], university students are more likely to be concerned about environmental issues and this makes them different from the rest of the population. Thus, future research should collect data from respondents that represent different backgrounds in various parts of the country. Second, the study was limited to the use of nonprobability sampling to gather respondents. Thus, future studies can be conducted using probability sampling to obtain nationally representative respondents. Third, the sample size was relatively small compared to the total population of China. Future research should thus aim to recruit thousands of respondents from different socioeconomic backgrounds to determine the generalizability of the study’s results. Fourth, although the two attitude items used in this study are reliable and provide a good reflection of respondents’ attitude towards GMF, we recognize that more items should be included to better capture such a construct. Thus, future endeavors should aim to develop a psychometrically validated measure of Chinese respondents’ attitude towards GMF. Finally, the study only examined attitude towards GMF without specifying whether the food comes from genetically modified plants or animals. Future studies should differentiate between these types of GMF considering that there are differences in attitude between these two sources of GMF [4].

## 8. Conclusions

Based on a sample of university students in Kunming, China, this study provides novel insights regarding attitude towards GMF as well as the significant personal and media factors related to them. Results revealed that the respondents had an overall negative attitude towards GMF. Regardless of age and gender, those with high food neophobia were likely to have a negative attitude towards GMF, whereas those that perceived greater GMF benefits were more likely to have a positive attitude towards it. Although media dependency was not associated with attitude towards GMF, a high level of attention to GMF-related media was associated with a negative attitude towards GMF. Overall, the study highlights that the overall negative attitude towards GMF among a sample of Chinese university students was not only influenced by personal factors (e.g., food neophobia and perceived benefit) but also by the level of media attention given by the respondents to messages conveying GMF-related information. We hope that such findings can guide scholars, practitioners, and government officials in developing messages to help improve the prevailing attitude towards GMF in China.

## Figures and Tables

**Table 1:** Regression results predicting attitude towards genetically modified foods (GMF).

	Zero-Order Correlation	*B*	SE	β
Block 1: Control variables				
Age	−0.16 **	−0.02	0.01	−0.08
Gender	−0.11 *	−0.08	0.06	−0.05
Incremental *R^2^* (%)				3.90 ***
Block 2: Personal factors				
Food neophobia	−0.11 *	−0.15	0.07	−0.09 *
Perceived benefit	0.55 ***	0.62	0.05	0.54 ***
Factual knowledge	−0.05	−0.02	0.02	−0.03
Perceived familiarity	0.05	0.06	0.04	0.07
Incremental *R^2^* (%)				28.30 ***
Block 3: Media factors				
Media attention	−0.04	−0.11	0.05	−0.09 *
Media dependency	0.06	0.02	0.05	0.02
Incremental *R^2^* (%)				0.40
Block 4: Two-way interactions				
Media dependency × media attention		−0.14	0.06	−0.09 *
Incremental *R^2^* (%)				0.80 *
Total R^2^ (%)				33.40 ***

Notes. *N* = 467. Cell entries for all models are final unstandardized (*B*) and standardized (β) regression coefficients for the final block; SE is standard error. * *p* < 0.05, ** *p* < 0.01, *** *p* < 0.001.

**Table 2:** Simple slope analysis results.

Level of Media Attention	*B*	SE	*p*	95% CI
−1 SD (−0.6399)	0.11	0.06	0.08	−0.01, 0.23
Mean (0.0000)	0.02	0.05	0.67	−0.08, 0.12
+1 SD (0.6399)	−0.06	0.06	0.31	−0.19, 0.06

Notes. *N* = 467. Only unstandardized (*B*) regression coefficients are produced by the program. CI is confidence interval; SE is standard error.
